# CD11b and TLR4 in human neutrophil priming by endotoxins from *Escherichia coli*

**DOI:** 10.1186/cc12974

**Published:** 2013-11-05

**Authors:** Isabella Prokhorenko, Dimitry Kabanov, Svetlana Zubova, Sergay Grachev

**Affiliations:** 1 Institute of Basic Biological Problems, Pushchino, Moscow Region, Russia

## Background

The interaction of endotoxins (lipopolysaccharides (LPS)) from Gram-negative bacteria with peripheral blood mononuclear cells leads to the assembly of a receptor cluster composed from mCD14, CD11b/CD18, TLR4, CD16A and CD36 [1,2]. It is well known that the main signal transducing receptor complex is TLR4/MD-2 while mCD14 is involved in the recognition of S or R endotoxin's glycoforms [3,4]. A growing body of evidence indicates that the CD11b/CD18 receptor plays the significant role in the endotoxin signaling machinery because it can influence TLR4-mediated cell activation [5]. So, using mAbs, we carried out experiments to elucidate the influence of CD11b inhibition on neutrophil priming by endotoxins for *N*-formyl-methionyl-leucyl-phenylalanine (fMLP)-induced respiratory burst.

## Materials and methods

**Figure 1:**
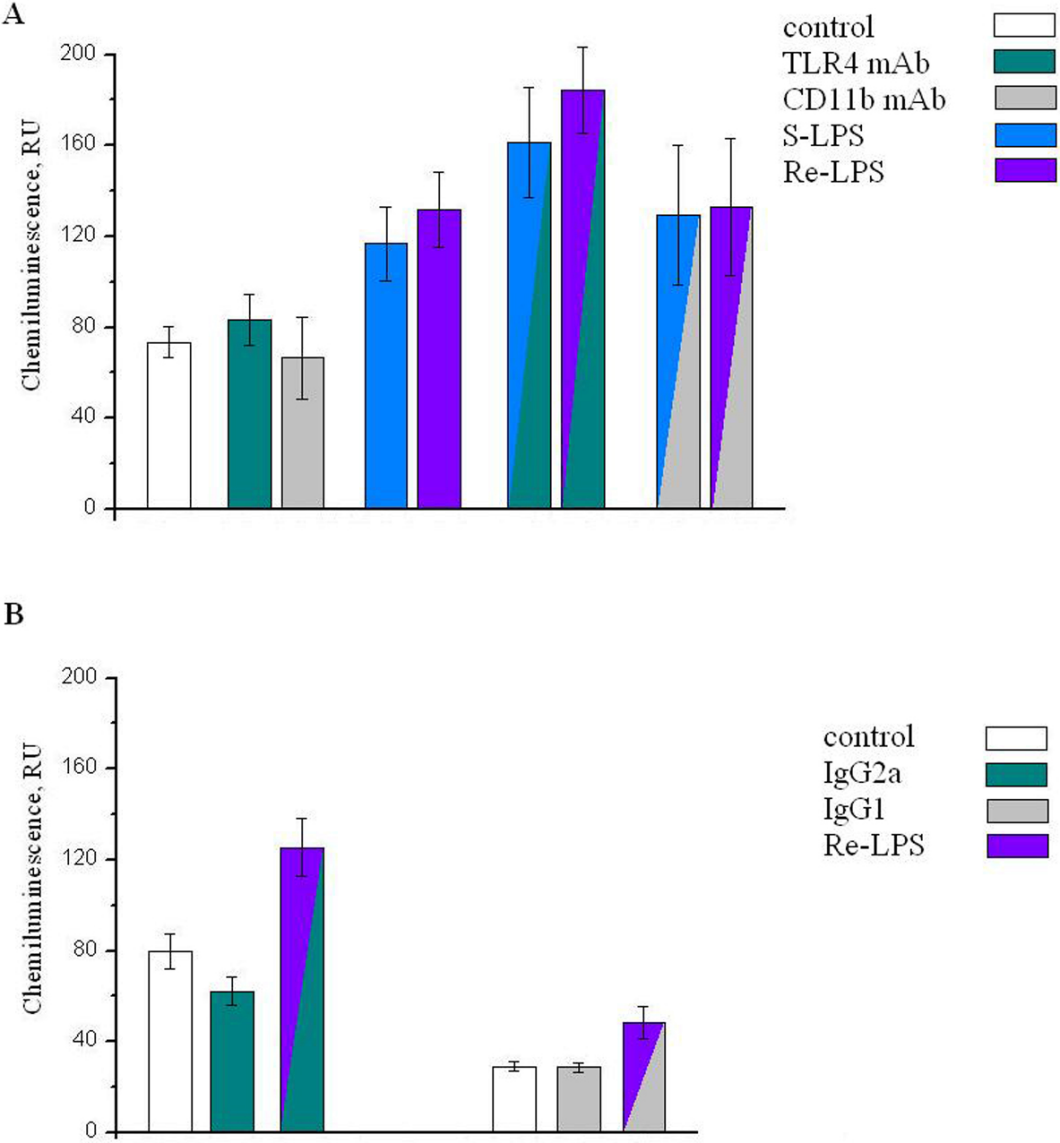


Human neutrophils were isolated from heparinized blood of healthy volunteers by standard procedure and incubated with or without anti-TLR4 mAbs (HTA125, IgG_2a_) or anti-CD11b mAbs (clone 44, IgG_1_) or isotypic immunoglobulin controls, respectively, for 30 minutes before stimulation with S-LPS or Re-LPS from *Escherichia coli *O55:B5 or JM103, respectively. The cells (2 × 10^5^), 2% of autologous serum, glucose and luminol in Ca^2+^-PBS buffer (pH 7.3), were placed in the chemiluminometer's chambers (37°C) and primed by S-LPS or Re-LPS (100 ng/ml) for 30 minutes (37°C). Reactive oxygen species (ROS) production was triggered by addition of fMLP (1 µM). The chemiluminescence reaction was monitored continuously for 7 minutes. Total ROS production by control and LPS primed neutrophils during the first 50 seconds after fMLP addition is presented in Figure 1.

## Results

Re-LPS revealed the most neutrophil priming activity in comparison with S-LPS (Figure 1A), which is in accordance with our previous work [6]. Actually, mAbs against TLR4 as well as against CD11b did not inhibit neutrophil priming by *E. coli *endotoxins. Moreover, the incubation of cells with anti-TLR4 or anti-CD11b mAbs followed by endotoxin priming increased fMLP-induced ROS production (Figure 1A). However, the differences between priming effectiveness of S-LPS and Re-LPS, which had been seen in endotoxin primed cells, were leveled by prior cell exposure to anti-CD11b mAbs. Neutrophils exposed to anti-TLR4 mAbs retained their ability to distinguish between S-LPS or Re-LPS being primed, respectively (Figure 1A). Incubation with isotypic IgG_2a _decreased fMLP-induced ROS production from unprimed neutrophils (Figure 1B) that was not observed in the case of IgG_1_. These results demonstrate that Fc regions of isotypic immunoglobulins and therefore of mAbs used in our study are not silent parts of these molecules regarding neutrophil surface receptors and their intracellular signaling pathways. Finally, the incubation of cells with isotypic immunoglobulins and then with Re-LPS did not abrogate neutrophil priming for subsequently fMLP-triggered ROS production.

## Conclusions

The inhibition of human neutrophil CD11b by specific mAbs (clone 44) did not abolish LPS-dependent neutrophil priming for fMLP-induced respiratory burst, but eliminated the capacity of these cells to distinguish between S-LPS or Re-LPS glycoforms. Unlike the effect of anti-CD11b mAbs, neutrophil exposition to anti-TLR4 mAbs (HTA125) did not inhibit neutrophil priming and capacity of these cells to distinguish endotoxin's glycoforms.
